# Tactile Interaction with Socially Assistive Robots for Children with Physical Disabilities

**DOI:** 10.3390/s25134215

**Published:** 2025-07-06

**Authors:** Leila Mouzehkesh Pirborj, Caroline Mills, Robert Gorkin, Karthick Thiyagarajan

**Affiliations:** 1 Smart Sensing and Robotics Laboratory (SensR Lab), Centre for Advanced Manufacturing Technology, Western Sydney University, Kingswood, NSW 2747, Australia; 19977775@student.westernsydney.edu.au; 2 Translational Health Research Institute, Western Sydney University, Westmead, NSW 2145, Australia; caroline.mills@westernsydney.edu.au (C.M.);; 3 School of Health Sciences, Western Sydney University, Penrith, NSW 2751, Australia

**Keywords:** robotic sensing technologies, inclusive healthcare robotics, tactile sensing, child–robot interaction, physical rehabilitation, rehabilitation technologies, tactile interaction, sensor applications

## Abstract

Children with physical disabilities are increasingly using socially assistive robots (SARs) as part of therapy to enhance motivation, engagement, enjoyment, and adherence. Research on SARs in rehabilitation has primarily focused on verbal and visual interaction, but little is known about tactile interaction (physical touch). The objective of this scoping review was to examine empirical studies published between 2010 and 2024 focusing on tactile interaction between SARs and children with physical disabilities, such as cerebral palsy (CP). Nine studies were identified as being eligible after a rigorous selection process, showing that although touch-based SAR interventions have been used in pediatric rehabilitation, structured methodologies and standardized tools are lacking for measuring tactile engagement. In light of the studies’ findings, it is evident that few studies evaluate the therapeutic effects of touch-sensitive SARs, underscoring the need for validated frameworks to assess their efficacy. In this review, SAR and tactile sensing researchers, rehabilitation specialists, and designers are given critical insights into how tactile interaction can enhance the role of SARs in physical therapy.

## 1. Introduction

Cerebral palsy (CP) is one of the most common physical disabilities originating in childhood. Globally, about 1.2% of children under five years of age are estimated to have CP, equating to roughly 8.1 million children. CP accounts for approximately 6.5% of the total years lived with disability among children under five, highlighting its significant contribution to global disability burdens. The prevalence is highest in low- and middle-income countries, where over 98% of affected children reside. Comparatively, intellectual disabilities affect around 2.4% of children under five [1,2]. This rate makes CP a significant contributor to global childhood disability, underscoring the importance of early intervention and rehabilitation efforts to support those affected, especially in resource-limited settings. CP is a disorder of posture and movement caused by an immature brain [3]. CP typically results from abnormal development or injury to the developing brain, most often occurring before birth. In some cases—particularly in preterm infants—this may involve underdeveloped brain structures or vulnerabilities associated with early gestational age. These are permanent disorders that affect movement and posture, causing limitations in activity, resulting from non-progressive changes in the developing fetal or infant brain [4]. Although perinatal care has advanced, the prevalence of CP has remained stable, underscoring the need for customized interventions. Early diagnosis is crucial to optimizing outcomes in children with CP [5]. Physiotherapy aims to improve balance, coordination of the limbs and body, motor control, and the active range of motion necessary to perform motor tasks [6].

**Figure 1:**
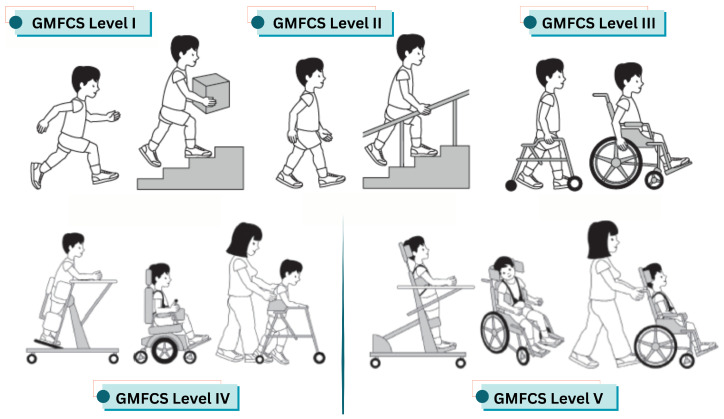
Overview of the GMFCS levels for children with cerebral palsy. Level I: Children can walk, run, and jump, but may have limitations in balance, coordination, and speed. Level II: Children walk in most settings but may require assistance or a handheld mobility device for long distances. Level III: Children use wheeled mobility for long distances and may self-propel for short distances. Level IV: Children typically rely on physical assistance or powered mobility to move around. Level V: Children are transported in a manual wheelchair in all settings [7].

The authors of [7] presented four standardized and reliable systems: the Gross Motor Function Classification System (GMFCS), the Manual Ability Classification System (MACS), the Communication Function Classification System (CFCS), and the Eating and Drinking Ability Classification System (EDACS). These systems facilitate clearer communication among care providers and more accurate subject stratification for research by providing a common language to describe the functional abilities of people with CP. The GMFCS, developed by CanChild in Canada, categorizes the gross motor skills (e.g., sitting and walking) of children and young people with cerebral palsy into five levels by examining movements such as sitting and walking, using a mobility device (GMFCS, Cerebral Palsy Alliance). Figure 1 illustrates how children with disabilities function at all five levels of the GMFCS.

There is significant potential for SARs, which are designed to assist through social interaction rather than physical interaction, to have a significant impact on pediatric therapy [8]. As opposed to general robotic systems, SARs are specifically designed to enhance motivation, engagement, and therapeutic outcomes through the use of social presence, touch, and communication, which increases motivation, engagement, and therapeutic outcomes [9]. This potential is especially relevant for children with physical disabilities, since SARs can be integrated into therapy in ways that encourage repeated participation [10]. Recent advances in assistive technologies emphasize their importance in physiotherapy, as they promote greater independence, improve quality of life, and support active participation in therapeutic interventions [11].

### 1.1. Social Robots in Pediatric Rehabilitation

Over the past decade, social robots have seen growing use in healthcare, particularly in pediatric rehabilitation. Despite the widespread adoption of other assistive technologies, robots are currently used by only 3.5% of children with disabilities [11]. Yet, the potential for robotic interventions to improve therapeutic outcomes is highly promising. Research has shown that physical agents tend to elicit stronger behavioral and emotional responses than virtual ones [12]. In pediatric rehabilitation, robots with human-like features can significantly enhance engagement, motivation, and overall therapeutic effectiveness. For example, a review by [13] highlighted the potential of SARs to improve motor skills, social interaction, and motivation in children with CP. Similarly, Ref. [14] identified three key benefits of robotics for children with CP: establishing neuromuscular pathways, increasing range of motion, and boosting motivation. Interestingly, children tend to accept and respond to social robots more readily than expected, although the reasons remain unclear [15].

SARs have proven useful in delivering exercise programs in pediatric rehabilitation [16]. Researchers have explored various scenarios, such as using robots to coach rehabilitation exercises [17]. Haptic feedback, in particular, has shown promise in improving motor learning performance and reducing errors [18]. However, tactile interaction in robotic systems is more commonly studied in the context of children with social interaction challenges, such as autism [19]. In these cases, touch is often used to address communication and social skill deficits [20,21,22,23,24]. To enhance child–robot interaction, researchers have employed both verbal and non-verbal behaviors. Among non-verbal cues, touch plays a vital role in emotional communication [25]. Physical contact fosters a sense of presence and connection [26], and interest in haptic communication within human–robot interaction (HRI) is growing [27]. Studies have explored how safe tactile interactions—such as handshakes or gentle touches—can influence human behavior [28,29]. These interactions can evoke a range of emotional, physiological, and behavioral responses, depending on the nature of the social relationship [30]. For robots to engage in meaningful social touch, they must be capable of both delivering and interpreting tactile input [26]. As interpersonal touch becomes increasingly rare in modern digital societies [31], researchers are investigating whether haptic technologies can fill this gap. Given the importance of non-verbal communication, it is essential to understand how robots’ non-verbal behaviors affect human perception and interaction [28,32,33,34,35]. For instance, Ref. [36] demonstrated that machine-generated touch can convey emotions that humans are able to interpret meaningfully.

In a study by [37], the researchers examined both physiological and subjective responses to tactile interactions with a humanoid robot. They found that changes in skin conductance—an indicator of emotional arousal—aligned with participants’ reactions to robotic touch. An early milestone in social-physical HRI was presented by [38], who found that people preferred warm, gentle robot hugs over cold, firm ones. This study suggested that robots could engage in familiar social gestures like hugs, high-fives, and dancing. Similarly, Ref. [39] reported that participants’ efforts improved when they received touch from a robot. Other studies have shown that robotic touch can reduce stress [40], increase generosity [41], and enhance perceptions of warmth and likability [42].

Understandably, children with certain medical conditions—such as leukemia—are often excluded from physical contact with robots due to sterility concerns [43]. However, with appropriate precautions, children with physical disabilities like cerebral palsy—who already engage with various therapeutic tools—could benefit from tactile interaction with robots during rehabilitation. Despite the growing interest in social robotics, few studies have examined the role of touch in therapeutic settings for children with physical disabilities. While humanoid robots have been used in this context [44,45,46,47], most interactions have been non-physical. For example, in [16], the robot primarily demonstrated exercises and provided verbal encouragement without physical contact. Similarly, Ref. [48] emphasized maintaining a safe distance between the robot and the child.

### 1.2. Taxonomy of Tactile Interaction in SARs

In the context of SARs, tactile interaction encompasses a variety of physical engagements between the user and the robot. However, the literature often uses terms such as tactile, haptic, and touch-based interactions interchangeably, which can lead to ambiguity. To provide conceptual clarity, this review adopts a taxonomy that distinguishes tactile interaction into two primary forms: affective touch and utilitarian touch. Affective touch refers to emotionally expressive, socially motivated contact such as hugging, stroking, or petting, which plays a role in emotional bonding and comfort [49]. In contrast, utilitarian touch encompasses goal-directed or function-driven actions such as button pressing, sensor tapping, and interface adjustment, where the focus is on task execution or system control [50,51]. This classification provides a framework for interpreting the diverse tactile behaviors reported in SAR studies and ensures consistent terminology throughout this review.

### 1.3. Research Questions of Our Study

Although SARs are increasingly used in rehabilitation settings, a significant knowledge gap remains regarding the impact of tactile interactions between robots and children with physical disabilities on rehabilitation outcomes. Most existing studies emphasize verbal or visual engagement, often overlooking the potential of touch to enhance therapeutic involvement and effectiveness. Furthermore, there is a lack of structured methodologies and validated tools to measure and evaluate tactile interactions in therapeutic contexts. Addressing this gap is crucial for developing SARs that can effectively leverage physical interaction to motivate and engage children during rehabilitation exercises while also supporting therapists in delivering therapy. In this review, we examine the literature obtained from multiple databases to explore the role of tactile interaction with SARs for children with physical disabilities. Our analysis focuses on categorizing the target users, types of robots, and forms of interaction. The findings reveal that only a limited number of studies—specifically nine—incorporated tactile interactions in their experiments, and only a small subset evaluated the effectiveness of touch for children with physical disabilities. Through this review, we aim to address the following research questions:

- Have previous studies involving SARs in therapeutic settings for children with physical disabilities (such as CP) incorporated tactile interaction?
- Is it possible to engage SARs and children with physical disabilities (such as CP) in meaningful touch-based interactions? In what ways does this affect the outcomes of therapy?
- How does the current literature describe and measure the duration, impact, and effectiveness of tactile interactions between SARs and children with physical disabilities?
- What are the key findings from the studies that included touch or tactile interactions with SARs in therapeutic settings?

### 1.4. Article Organization

The remainder of this manuscript is structured as follows. Section 2 outlines the methodology, including the inclusion and exclusion criteria; the literature search strategy; and the data-extraction procedures. Section 3 presents the results of the publication search across various databases and analyzes the eligible studies, focusing on the types of robots used, participant characteristics, and the evaluation methods and metrics employed. Section 4 discusses key themes such as the material safety and appearance of SARs, tactile-based interactions, the essential components identified in the reviewed studies, and the highlights of significant findings. Finally, Section 5 summarizes the studies’ outcomes and offers recommendations for HRI researchers and designers of social robotic systems.

## 2. Methodology

In this section, the methodology adopted in this scoping review, as well as the criteria for inclusion and exclusion, are described.

### 2.1. Inclusion and Exclusion Criteria

The following criteria guided our selection of studies focusing specifically on tactile interaction between SARs and children with physical disabilities:

- We reviewed several databases covering the period from 2010 to 2024. This timeframe was chosen because assistive technology, particularly social robots, has seen significant advancements over the past decade. To ensure comprehensive coverage, we also included Google Scholar. This approach allowed us to include a broader range of studies without excluding other databases.
- All robot types were explicitly limited to SARs, which include humanoid robots, animal-like robots, or socially interactive robots. Since the primary aim of this review was to explore the interaction aspects of robots, studies employing non-social robots, such as arm robots, industrial robots, exoskeletons, or wearable robotic technologies, were excluded.
- We included studies involving children with physical disabilities (aged 18 months–16), excluding those involving children without disabilities. In pediatric rehabilitation research, this age range (18 months–16 years) represents a broad range that is suitable for studying physical interactions with SARs. Our focus was on children with physical disabilities such as CP, not on those with mental and cognitive impairments like Autism Spectrum Disorder (ASD) or Attention Deficit Hyperactivity Disorder (ADHD). Studies involving children who are cancer patients without cognitive impairments were included, as the robot tasks and duties are applicable to children with physical disabilities like CP.
- Only empirical studies were included; reviews, ethical discussions, and theoretical discussions were excluded.
- We specifically focused on studies that involved tactile situations (physical interaction) between children and SARs. Studies that included children with physical disabilities and SARs but did not involve tactile interaction were excluded.
- Articles published in English in journals and conference proceedings (excluding theses) available through electronic abstract systems were used.

### 2.2. Literature Search Strategy

Based on our research objectives, we developed search terms that focused explicitly on touch-based interactions with children with disabilities and socially assistive robots. In the search string, we used the following keywords: (“humanoid robots” OR “social robots” OR “child–robot interaction”) AND (“tactile interaction” OR “haptic feedback” OR “touch interaction” OR “physical interaction” OR “tactile sensor”) AND (“child” OR “young” OR “teenager”) AND (“disable*” OR “special needs” OR “cerebral palsy”) AND (“rehabilitation sessions” OR “therapy sessions”). To ensure broad coverage of disability-related terms, we used the wildcard disable*, which captures variations such as disabled, disability, and disabilities. Additionally, we explicitly included terms such as “physical disability” and “physical disabilities” to enhance specificity and comprehensiveness.

To identify relevant studies on tactile interaction between humanoid robots and children with disabilities, we conducted a comprehensive search across four major academic databases: IEEE Xplore, Google Scholar, the ACM Digital Library, and Springer. These platforms were selected for their broad coverage of research on robotics, rehabilitation, and related fields. IEEE Xplore provides access to peer-reviewed journal articles, conference proceedings, and technical standards in computer science, electrical engineering, and allied disciplines. The ACM Digital Library offers a full-text collection of ACM publications and a comprehensive bibliographic database focused on computing. Springer is a global publisher of journals, books, and conference proceedings in science, technology, and medicine, including many works relevant to robotics and healthcare. Google Scholar was included to ensure broader coverage, as it indexes the scholarly literature across various disciplines and publishing formats, including content from IEEE, Springer, ACM, and other academic sources. The search results from Google Scholar included articles and conference papers published by leading publishers such as IEEE, Springer, MDPI, ACM, Elsevier, Taylor & Francis, Frontiers, SAGE, Wiley, JMIR Publications, De Gruyter, and others. We searched for both journal articles and conference proceedings across all platforms to ensure a thorough and inclusive review of the literature. The results of each search are presented in Section 3.1.

### 2.3. Data Extraction for Preparing the Primary Literature

The first author screened all titles and abstracts after conducting the database searches to identify potentially eligible articles. As a result, the first author reviewed the complete texts of the selected studies and extracted relevant data using a structured approach to ensure consistency across studies. The remaining three authors independently reviewed the extracted data to ensure they were accurate, consistent, and objective in their interpretations.

## 3. Results

### 3.1. Search Results

In order to conduct a comprehensive literature search, we searched four major databases: the ACM Digital Library, IEEE Xplore, Springer, and Google Scholar. Our search was performed in April 2025 using the keywords provided in the previous section.

Table 1 presents the sources used for searching publications, the time periods covered, and the number of papers published.

For the period 2010–2024, we found 60 publications in the ACM Digital Library, 0 in IEEE Xplore, and 103 in Springer. Additionally, we conducted a search on Google Scholar, as it indexes a wide range of publishers and databases. Our search yielded a total of 69 publications. These included 9 from IEEE, 21 from Springer, 6 from MDPI, 8 from ACM, 5 from Elsevier, 5 from Taylor & Francis, 2 from Frontiers, 2 from SAGE, 1 from Wiley, 1 from De Gruyter, and 1 from JMIR Publications. The remaining eight publications were sourced from various other bibliographic databases. It is important to note that this count may contain duplicate entries across databases.

### 3.2. Reporting on Robot and Participant Information in Eligible Publications

**Figure 2:**
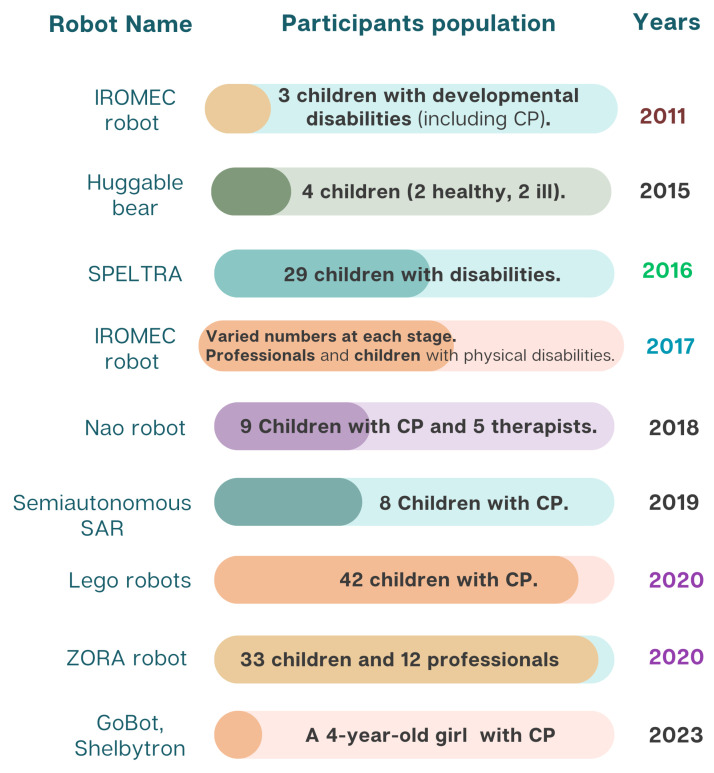
Robot names, participant information, and publication years of eligible studies.

From the various database searches, only nine studies met the eligibility criteria upon review. Therefore, this review focuses on nine empirical studies that explored the use of SARs in therapeutic settings for children with physical disabilities. Figure 2 summarizes the names of the robots, the population and health information of the participants, and the year each study was published.

Based on the data in Figure 2, the sample sizes across the studies varied significantly, even within the same year. For example, in 2020, one study involved 42 children [52], while another involved 33 [53]. In contrast, the smallest studies involved only three participants, one in 2011 [54] and one in 2023 [55]. Other studies, such as [56], had moderate sample sizes (e.g., eight children in 2019).

One study on the IROMEC robot [57] followed a multi-phase approach involving professionals and caregivers, although the number of children involved was not specified. The variation in sample sizes reflects differences in study design and the practical challenges of recruiting children with physical disabilities for such interventions [58].

### 3.3. Methods and Measurements Used in Studies

It is important to combine qualitative, observational, and physiological measurements in the assessment of tactile interactions between SARs and children with physical disabilities. In Table 2, we describe how interactions were observed, recorded, and evaluated in the eligible studies. Researchers employed behavioral, self-report, and physiological measures to investigate human–robot touch-based interactions. There are, however, a number of challenges associated with tactile HRI research, such as the lack of standardized methodologies, which makes it difficult to compare findings across studies.

Research has shown that touch-based interactions foster prosocial behavior and engagement in HRI [59]. Several studies have found that motor mimicry and affective touch contribute to improving human–robot relationships, while others have found that robot-initiated touch alone is less likely to invoke physiological or emotional responses [32]. A review of tactile sensing techniques discussed various methods based on sensor placement, covers, and sensitivity, and illustrated how these factors affect robot behavior and sensory accuracy [60]. Despite these advancements, most studies on SARs in rehabilitation do not employ physiological tools like Galvanic Skin Response (GSR), heart rate variability (HRV), or skin conductance response (SCR) to measure the effects of tactile engagement.

It is important to mention that Table 3 includes all measurements used in each study to evaluate interactions between SARs and children, not just those focusing on tactile or touch-based interactions. This is because in each of the nine eligible articles, the experiments facilitated sufficient closeness between children and SARs to allow for the possibility of tactile interaction. However, not all studies measured this type of interaction (tactile) if it occurred. One important reason for this may be that the target group had physical limitations that may have prevented them from successfully reaching objects (social robots).

While our review focuses on studies involving children with physical disabilities, we also reference research conducted with healthy adult participants to highlight established methods for evaluating tactile interaction. These studies offer validated measurement tools and methodological frameworks that are currently underutilized in pediatric rehabilitation contexts. It is important to note that in the reviewed studies involving children, tactile interaction typically forms only one component of a broader therapeutic intervention. This contrast underscores a critical gap in the literature and suggests an opportunity to adapt and apply these robust tactile assessment techniques to enhance therapeutic outcomes for children with physical disabilities.

**Table 2:** Summary of tools, measurements, and interaction situations in eligible articles.

Ref.	Study Objectives	Measurement Method and Tools	Interaction Description
[52]	To improve hand performance while playing with robot.	Kinematic analysis using camera and Finger tapping test.	To manipulate the puppets, children used touch sensors on arms and ultrasonic sensors on legs.
[53]	(1) To evaluate the contribution of the robot ZORA in supporting therapeutic and educational goals within rehabilitation and special education for children with severe physical disabilities, and (2) explore the roles that professionals attribute to ZORA during robot-assisted play interventions in these settings.	The assessment tools used included the Individually Prioritized Problem Assessment (IPPA), the Visual Analogue Playfulness Scale, and video-stimulated recall interviews. To help children express their feelings, a smiley scale was also employed.	Children interacted directly with ZORA by touching specific parts of its body—its hands, feet, and head. In response, ZORA performed corresponding actions: it shook hands when its hand was touched, gave a high five when its foot was touched, and waved when its head was touched.
[54]	To evaluate the short-term effects of using the IROMEC robot toy to support play during occupational therapy interventions for children with developmental disabilities.	Playfulness and engagement are measured with the Test of Playfulness (ToP 4.0). Using a 10-point Visual Analogue Scale (VAS), therapists provide feedback. Occupational therapists’ perspectives on usability and value added.	Children interacted with the IROMEC robot using three control methods: colored buttons, touchscreens, and body-guided motions. During play scenarios, they navigated space, engaged in pretend play, and used buttons to interact with the robot.
[55]	To explore how assistive robot help children with motor disabilities.	Child movement and engagement were assessed using video coding, sensor data, overhead tracking, a 30-second walk test, and self-report questionnaires, including Negative Attitude Towards Robot Scale (NARS) and the Trust Perception Scale-HRI. Child-robot spacing was also analyzed.	Children with motor disabilities used the Body-Weight Support System (BWSS) for overground mobility and were encouraged to follow and engage with the robot through close physical proximity and touch.
[56]	To compare SARS with a switch-adapted toy in assessing engagement levels and changes.	Visual Regard: Each session was recorded using two front-facing cameras (A child watches or tracks a robot). Child reaches for the robot (unilateral/bilateral). Fine Motor Movements: These included grasping the SAR or toy.	Playful interactions were designed to engage the child with the SAR. Although the robot was positioned within the child’s visual field, its movements and sounds were influenced by the child’s interactions with it.
[57]	To explore the potential of using the IROMEC robot in rehabilitation and special education for children with severe physical disabilities.	Individual Interviews (Conducted with therapists and special educators), Focus Group Interviews (Two Rounds), Digital Questionnaire (After showing a demo video). Not direct child measures (Future research phase).	Turn taking, sensory reward, make it move, follow-me, get in contact; using buttons/touch screen.
[61]	To explore the development of the humanoid social robot NAO as a socially assistive rehabilitation aid for children with cerebral palsy, with a strong emphasis on stakeholder engagement and on-site development.	Observation notes during sessions, surveys of parents, therapists, and patients, observation logs, and Heerink’s robot acceptance questionnaire.	NAO’s head-based sensors offer a tactile interface. A single tap moves to the next activity or starts repetitions, while double taps after a sustained press adjust the exercise speed.
[62]	To develop a low-cost robotic assistant capable of supporting a range of activities conducted during speech-language therapy sessions for children with various disabilities.	Scores from phonological, semantic, morphosyntactic exercises, and interaction levels. Researchers compared robot-assisted therapy (labeled "R") with manual therapy (labeled "M").	Games that encourage tactile engagement require touch input via the screen on the robot’s belly. Playing the games and completing the exercises requires children to touch the robot’s belly.
[63]	To design the Huggable robot to engage children through playful interaction and provide socio-emotional support within pediatric care settings.	Observations included interactions, analysis of various physical touch, and verbal responses. Identify and classify types of touch (e.g., hugging, petting, high-fiving). Frequency of touch (Healthy vs. Ill Children).	Robots were hugged, tickled, petted, and given high-fives as indications of physical contact. Robots were perceived as peers, and children conversed with them like other children—narrating experiences, showing personal items.

**Table 3:** Study assessment types and outcome measures.

Ref.	Measurement/Behavior	Assessment Type
[52]	Range of Motion Movement Speed Finger Tapping Test Pinch Strength (Jamar hydraulic pinch gauge) MANOVA/ANCOVA results Parent/Caregiver Perceptions (interviews) Therapist/Researcher Observations Adherence /Completion Rates	Objective Objective Objective Objective Objective Subjective Subjective Subjective
[53]	Individually Prioritized Problem Assessment Children’s Feelings (Smileys) Qualitative Interviews Session Notes Video Recording	Objective Subjective Subjective Subjective Objective or Subjective
[54]	Test of Playfulness IROMEC Evaluation Questionnaire Scores Statistical Analysis (Wilcoxon Signed Rank Test) Session Frequency and Duration Therapist Goal Achievement Ratings Therapist Evaluation of Robot Child Behavioral Observations	Objective Objective Objective Objective Subjective Subjective Subjective
[55]	Self-Initiated Steps Ankle Movement Counts Overground Movement Distance 30-Second Walk Test Distance Child-Robot Interaction Child-Robot Spacing BWSS Use Duration Parent Surveys (Engagement) Clinician/Parent Observations	Objective Objective Objective Objective Objective Objective Objective Subjective Subjective
[56]	Visual Regard Vocalization Reach Grasp Pulling Away Emotion (Positive/Negative)	Objective Objective Objective Objective Subjective
[57]	Questionnaire Response Rates Goal Identification Frequencies Professional Experience Range Expert Opinions on Robot Suitability Focus Group Discussions and Consensus Spontaneous Reactions to Robot Demonstration Improvement Suggestions Meta-plan Methodology Outcomes	Objective Objective Objective Subjective Subjective Subjective Subjective Subjective
[61]	Session Completion & Exercise Log System Performance & Failures Configuration Time & Interface Use Patient Interaction & Task Engagement Survey Responses Open-Ended Feedback	Objective Objective Objective Subjective Subjective Subjective
[62]	Exercise Scores Error Rates Session Completion Rates Interaction Level Parental Involvement and Feedback Therapist Observations	Objective Objective Objective Subjective Subjective Subjective
[63]	Physical Contact Frequency Touch Type Classification Verbal Response Frequency Interaction Duration Child Enjoyment Caring and Empathetic Behaviors Emotional Response to Session End Peer-like Perception Animal-like Interaction Behaviors	Objective Objective Objective Objective Subjective Subjective Subjective Subjective Subjective

The diversity in measurement approaches likely reflects the varied research objectives, experimental designs, and study contexts. To enhance understanding, we present the methods and measurements from four additional studies (not eligible for final consideration) involving participants without disabilities. Even though these studies did not meet the inclusion criteria for this review, their measurement approaches provide insights into potential methodologies for assessing tactile engagement in children’s SAR-based rehabilitation.

In the study by [30] involving participants without disabilities, the following touch-based interaction measurements were performed:

- Physiological measurements: GSR, heart rate (HR), heart rate variability (HRV; operationalized by the root mean square of successive differences), and respiration rate.
- Self-report measures: Valence and Arousal (Self-Assessment Manikin), Positive and Negative Affect Schedule, Fear Arousal Scale, Disgust Arousal Scale, Perceived Social Closeness (Inclusion of Other in the Self), and Robot Likability and Anthropomorphism (Godspeed questionnaire).

In [37], the authors discussed physiological measurements such as SCR. Subjective measurements included perceived impressions of the interaction (friendliness, comfort, arousal), assessed via a questionnaire and the NARS. In the study by [39], objective measurements included the number of actions completed in the task and the amount of time spent on the task (working time). Subjective measurements included a questionnaire assessing the perceived friendliness of the robot on a 7-point scale. In the study by [64], objective measurements of the interaction were discussed. A video recording captured participants’ immediate reactions to touch, including various coded behaviors such as gazing at the touched hand, smiling, raising eyebrows, and prosocial behavior. The following interaction measurements were performed:

- Subjective measurements: These included robot attitudes, robot anxiety, and prior experience questionnaires. The robot’s touch was assessed using the Relational Communication Scale, which evaluates the touch experience based on semantic differentials (only for touch conditions). The Inclusion of Other in the Self Scale was used to assess perceived closeness.
- Physiological measurements: SCR.

Based on the comparison, it was evident that the four non-eligible articles (which did not focus on children with disabilities) analyzed touch and tactile interactions using more detailed and diverse measurement tools and strategies. Most studies focused on observing child–robot interactions, often using “reaching out” to social robots as motivators or to increase engagement, rather than quantifying tactile duration, repetition, and emotional effectiveness. In these four articles, physiological tools such as GSR, HR, HRV, and SCR provided objective and quantitative insights into participants’ emotional and arousal states. These tools allowed for the analysis of participants’ physiological reactions to touch. In contrast, nine of the included articles lacked or underutilized such tools, placing more emphasis on qualitative or observational methods. The four excluded studies, conducted on participants without disabilities, emphasized tactile interaction using tools like the Relational Communication Scale and semantic differentials to assess touch perception. However, the nine eligible articles involving participants with disabilities used behaviors (e.g., physical gestures, task performance) rather than specific tools to evaluate touch or tactile interactions.

There is a need to develop standardized methodologies that combine behavioral observations with physiological and self-report measures in order to evaluate tactile interactions in SARs more effectively.

## 4. Discussion

Previous systematic reviews have shown that robots are effective in rehabilitation and education for children with disabilities, particularly those with ASD and CP. However, these reviews also emphasized the need for clinical research and user-centered design approaches [65]. Meta-analyses have demonstrated that SARs effectively engage children with CP in therapeutic interventions, but further development is needed in areas such as aesthetics, functionality, therapeutic programming, and success metrics [66]. According to [13], most studies in this field rely on small sample sizes, involve only single-session exposures, and do not include validated outcome measures. There is a need for extended research and long-term evaluations to fully investigate the therapeutic potential of SARs. Additionally, Ref. [67] found that telepresence robots exhibit limitations in both accessibility and evaluation practices. Based on these findings, our review examines touch and tactile interaction in SARs research for children with physical disabilities. We analyzed nine empirical studies and identified a significant gap in understanding the impact and effectiveness of touch-based interactions on communication between SARs and children with physical disabilities. This aspect of HRI (specifically child–robot-interaction) has not been previously addressed in pediatric rehabilitation settings, as earlier reviews did not specifically focus on this aspect for children with disabilities.

### 4.1. Safety and Appearance of Therapeutic Robots

For pediatric rehabilitation, particularly for children with physical disabilities such as CP, the design and materials of humanoid robots are crucial for facilitating effective tactile interactions. Studies that used humanoid/social robots in pediatric rehabilitation without tactile interaction mostly employed commercial robots such as NAO [16,68,69,70]. However, this does not mean that current commercial SARs cannot be used to include and evaluate tactile interactions, as demonstrated in [53,61]. The most popular use of SARs in pediatric sessions is as a coach, feedback provider, and motivator [48,71,72]. These tasks typically involve a distance between children and robots in the experimental setup. Despite the popularity of commercial humanoid robots like NAO or Pepper in therapeutic settings due to their advanced interactive capabilities, their design often includes harder materials, posing challenges for direct tactile interactions with children with physical disabilities. This issue is particularly acute for interactions involving close proximity and possible physical contact. Among the nine eligible studies, two robots represented humanoids (ZORA, based on the NAO platform, and a standalone NAO) [53,61], four robots were inspired by animals (the huggable teddy-bear robot, SPELTRA bear-like robot [62], Lego puppet robot [52] with animal costumes, and the Shelbytron/GoBot robot—one of which resembles a dog–fox hybrid) [55], and three robots were non-humanoid mobile platforms (IROMEC, the generic semi-autonomous SAR in [56,57], and GoBot’s sister platform without animal styling). It is not just about aesthetics: softer outer shells (such as plush fabric on the huggable robot and EVA foam on the SPELTRA robot) were introduced specifically to enhance safety by reducing sharp edges and protecting fragile pediatric skin and limbs.

Further, studies on the IROMEC robot provide insight into how materials used in robot design affect tactile engagement with SARs [54,57]. The IROMEC robot is a gaming and rehabilitation platform developed within the FP6 project to support children with severe physical disabilities. The researchers acknowledged the robot’s effectiveness at promoting play and engagement [57]; however, several practical limitations were highlighted, such as the hard plastic body, neutral color scheme, fixed emotional expressions, and small buttons. It was argued that a platform with softer wipe-clean covers, brighter exchangeable costumes, clearer facial displays, and larger or voice-activated controls would be safer, easier to access, and more engaging for children with severe motor impairments. While IROMEC offers children with severe motor impairments the ability to interact physically with buttons, screens, and movement tracking, its physical structure lacks the adaptability they need [54].

**Figure 3:**
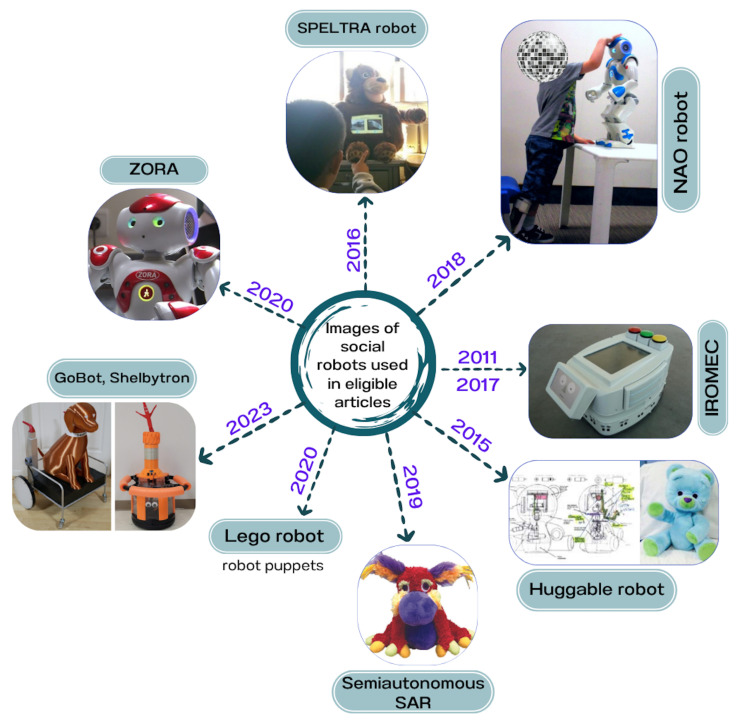
Robots from the eligible studies: Semi-autonomous SAR [56], Lego robot [52], GoBot/Shelbytron [55], ZORA [53], NAO [61], SPELTRA robot [62], huggable robot [63], IROMEC (2017) [57], IROMEC (2011) [54]. This figure illustrates the chronological development and visual appearance of the robots in pediatric rehabilitation.

In [52,55,56,62,63,73], the researchers showed that when therapeutic activities require close interactions, they often design and build custom robots to meet safety needs. Nevertheless, the high cost of commercial robots remains a consideration in designing robots. Figure 3 displays images of the robots featured in the nine eligible articles, along with the years in which these experiments were conducted.

### 4.2. Tactile-Based Child–Robot Interaction Studies in SARs

There is an interesting trend in SAR studies involving touch and tactile interactions, particularly when examining different groups of children with special needs. Most interactions centered around touch and physical interactions target autistic children [74,75,76]. Several factors contribute to this focus on autism, including sensory processing differences. Studies such as [74,77] provide valuable insights into the role of touch in HRI, but they do not address the specific challenges faced by children with physical disabilities in rehabilitation settings.

In a few studies, however, tactile-based interventions have been shown to have therapeutic potential for children with developmental and motor impairments. As an example, CARBO, a robot that facilitates tactile engagement for children with developmental disorders, has shown promising results in addressing sensorimotor impairments through interactive games [78]. A similar study on affective touch in HRI supports tactile-based SAR interventions in pediatric rehabilitation by enhancing user engagement and trust through haptic feedback mechanisms [79]. SARs also benefit from the use of tactile sensor technology, which reveals how advanced haptic interfaces and sensorized artificial skin can facilitate more natural and adaptive physical interaction [80]. In affective HRI, robotic touch has also been explored, demonstrating that touch-based interventions can help regulate emotions and influence physiological responses, which could have significant implications for rehabilitation therapy in children with disabilities [81]. The use of tactile interaction in SARs has been extensively studied in autism studies; however, there is little research examining how it can enhance motor function, engagement, and rehabilitation outcomes for children with CP.

### 4.3. Tactile Interaction Analyses in the Eligible Papers

Children’s touch-based interactions with robots were frequently reported in the studies reviewed, but their outcomes were usually not measured. Only several studies (four out of nine) directly focused on the impact of touch-based interactions. Physical touch and interaction types were explicitly examined in the huggable robot study [63], in which tactile engagement was shown to facilitate both socio-emotional and physical interactions. The huggable robot [63], with its child-friendly and furry appearance, can perform swift and smooth motions. As a result of the huggable robot’s physical touch encouragement and response, children felt a strong emotional connection and engagement with it.

Studies involving custom robots, like GoBot and Shelbytron [55], NAO [61], SPELTRA [62], and the robot designed in [56], reported close physical proximity and touch potential but did not typically focus their measurement strategies on tactile interactions. For example, in [56], the robot was placed in the child’s visual field, close enough for the child to touch it, but the robot’s movements and sounds primarily prompted the interaction. The study focused on children’s interaction with SARs and switch-adapted toys, which were activated by children touching a red button [56].

Although the NAO robot study [61] did not emphasize direct physical contact, the tactile interface was used for interaction. For example, tapping once moved to the next activity or began repetitions, and long-pressing the front and rear buttons paused the NAO robot. The study aimed to adapt the robot for rehabilitation use, with touch-based interactions designed primarily as control and interaction tools rather than specifically therapeutic ones.

Even though SPELTRA [62] used a touchscreen for interaction on the bear robot’s belly, tactile engagement was still involved. In the SPELTRA robot study, the touchscreen served primarily as an interface for interaction and visualization rather than as a therapeutic device. In the study involving the BWSS in [55], the researchers did not explicitly design the robot for touch-based interactions but measured the distance between the robot and the child, which may have functioned as a proxy for potential touch-based interactions.

In the ZORA study [53], touch-based interactions were incorporated into the robot’s design, allowing children to shake hands, give high-fives, and touch the robot’s head, which initiated responses from the robot. This demonstrates a structured approach to using touch in therapeutic and educational settings. However, detailed measurements or analyses of these tactile interactions were not provided [53]. In comparison, the Lego robot study [52] utilized touch sensors to control the puppet’s limbs, integrating touch as a functional element of the interaction but not as a primary measurement focus.

A number of tactile interactions were incorporated within the IROMEC robot studies [54,57] in order to provide assistance to children with severe physical disabilities through rehabilitation and educational play interventions. Interactive color buttons, a digital touchscreen, and direct physical manipulation are all tactile possibilities offered by IROMEC. The robot’s interactive play scenarios, such as “turns,” “sensory rewards,” “make it move,” and “follow me,” explicitly encourage physical engagement. Yet the authors stressed the need for improved adaptability, stability, and enhanced technical design to better meet children’s individual needs.

Table 2 presents further details on the interaction scenarios between children and robots, including the nature of physical interactions. However, in most of the reviewed studies, tactile interactions were either not measured or not analyzed.

### 4.4. Robots, Sensors, and System Components

Developing SARs and therapeutic robots for children with physical disabilities requires advanced sensors and system components. Children with disabilities benefit from these technologies in terms of physical interaction, responsiveness, and therapeutic engagement [82]. The authors of [60] discussed appropriate sensors for use within their identified HRI categories, categorizing tactile sensing techniques into three types based on sensor coverings: a hard shell, a flexible substrate, or no covering. This section briefly mentions how integrating sensors and devices into assistive robots enhances child–robot interaction (CRI) and facilitates therapeutic outcomes, as seen in the nine eligible articles.

The system in [55] monitors and analyzes movement during therapy sessions using a body-weight support harness system, ActiGraph GT9X-BT Link Activity Monitor (ActiGraph Corp, Florida, USA) sensors, and GoPro Hero Black 10 (GoPro, USA) cameras. The Shelbytron robot is controlled by a Teensy 3.6 microcontroller, and the GoBot robot operates on a TurtleBot2 base running ROS Noetic in Ubuntu 20.04 on a Raspberry Pi 4. This robot is teleoperated and uses light, music, and inflatable air-dancer stimuli.

The NAO robot in [61] features a tactile interface that enables children to interact through single, double, or long button presses, facilitating various interaction modes. The ZORA robot [53], built on the same platform as the NAO robot, has programmable sensors on its feet, hands, and head.

SPELTRA [62] uses a Raspberry Pi Model 2 B+, a 5MP camera for face recognition, a microphone for speech recognition, and a resistive touchscreen for user interaction. It also has text-to-speech capabilities for auditory feedback. The huggable robot in [63] uses internal sensors with Android smartphones to calculate and read data, with 12 capacitive-touch sensors around the body and pressure sensors in the paws to improve tactile responsiveness. The phone communicates with the motor boards using SparkFun’s IOIO board for Android to control the robot’s physical joint movements.

The puppet robots in [52] are equipped with ultrasonic and touch sensors, allowing for interactive puppetry and movement, enabling children to manipulate the robots through touch.

The SAR in [56] is built on an m3pi hobbyist robot platform, operating with wireless Xbox controllers and SONAR sensors for local environmental sensing. Microsoft Kinect 2 is integrated into the system to gather spatial information about a child’s movements.

A mobile, modular robot called IROMEC [54,57] was designed specifically for playing and facilitating therapeutic activities with children with physical and developmental disabilities. The robot features 13 ultrasonic sensors, 18 infrared sensors, and a laser scanner for safe navigation through therapeutic environments. An integrated motor/gearbox/encoder assembly ensures smooth interactions tailored to children’s abilities by precisely controlling the robot’s speed and movement. It also features two interactive touchscreens: an 8-inch head screen that displays facial expressions, and a 13-inch body screen that displays playful graphical elements. Play-based therapy environments benefit from IROMEC’s diverse sensor systems and interaction modes in order to promote children’s engagement, motivation, and functional development.

In Table 4, the information reported in the nine eligible studies is aggregated by robot platform (i.e., NAO and ZORA, which share the same hardware and therefore should be grouped).

These sensors and devices enable robots to respond dynamically to the needs and actions of children with physical disabilities in therapeutic settings.

### 4.5. Summary of Key Findings in Eligible Publications

This section highlights the key findings in the nine eligible articles on the role of SARs in improving interactions and therapeutic outcomes for children with disabilities.

In the study by [55], assistive robots (Shelbytron, GoBot) were found to be effective in maintaining children’s engagement with the BWSS. The engaging stimuli from these robots, such as lights, sounds, and interactive features, encouraged children to interact and move toward them. Parents rated their child’s excitement, enjoyment, interaction, and safety in the harness as high during most sessions.

The authors of [61] adapted the NAO robot as a socially inclusive rehabilitation aid through a two-phase design process that involved children, parents, and rehabilitation professionals. Physiotherapists and parents generally found the robot to be useful and easy to use in rehabilitation therapy.

The authors of [62] developed SPELTRA, a low-cost robotic assistant for speech therapy for children with disabilities. While there was no significant difference in the results between robot-assisted and manual therapies in the morphosyntactic area, robot-assisted therapy showed more consistent outcomes. Both manual and robot-assisted therapies yielded similar results in semantics, with a slight advantage for manual therapy.

The authors of [63] designed a huggable robot with a friendly and non-threatening appearance for children in hospitals. Haptic sensors allowed the robot to respond to physical touch from children. The study found that children with medical conditions had significant physical contact with the robot and responded positively, indicating that touch plays a crucial role in child–robot interactions.

The authors of [52] found that an intervention using Lego robot-adapted Chinese puppets significantly improved hand function in children with CP. The vibrant colors, varied textures, and interactive elements of the Lego robot puppets increased children’s interest and motivation in therapy. The intervention group showed more consistent improvements in finger movement speed and pressing performance compared to the control group.

The authors of [53] discussed how the ZORA robot interventions significantly contributed to reaching children’s individual goals, particularly in movement and communication skills. Professionals noted that ZORA played an important role as a motivator, rewarder, and instructor, helping children concentrate during sessions. However, technical and practical challenges, such as the need for proper training for professionals, were identified.

The authors of [56] compared a semi-autonomous SAR with a switch-adapted toy for children with complex cerebral palsy. Five of eight subjects engaged more positively with the SAR, showing more frequent and varied responses, including vocalizations and active engagement, compared to the switch-adapted toy, which required direct prompting by the investigator. The study suggested that SAR technology could enhance clinical interventions for children with complex cerebral palsy.

The authors of [57] demonstrated that the IROMEC robot could significantly improve motivation, social interactions, movement functions, learning, and communication in children with severe physical disabilities. Special educators and therapists reported that IROMEC was beneficial for promoting playfulness and engagement in therapy and education. A large-scale application, however, was considered unfeasible due to limitations regarding adaptability and technical stability.

The authors of [54] showed that IROMEC had a beneficial effect on playfulness and functional behavior in children with developmental disabilities, especially those with cerebral palsy. In spite of the clinical relevance of changes in playfulness, the sample size limited statistical significance. Therapy professionals and children were positive about the robot, but therapists suggested that with more technical refinement, the robot would provide more therapeutic value.

SARs proved to be effective in pediatric rehabilitation settings across all nine eligible studies reviewed. Through close and tactile interactions with the robots, children with physical disabilities improved engagement, motivation, playfulness, social interaction, communication skills, motor performance, and emotional responses. However, only three of the nine eligible studies [52,63] directly discussed the role and effectiveness of tactile interaction, despite all articles providing opportunities for physical interaction through touch.

### 4.6. Study Limitations

Despite the comprehensive scope of this review, several limitations should be acknowledged. First, more than half of the included studies involved small sample sizes, which limits the generalizability of their findings. This is a common issue in early-stage research on SARs, particularly in pediatric rehabilitation contexts.

Second, the lack of standardized and validated tools for assessing tactile interaction presents a significant challenge. This inconsistency hinders the ability to synthesize findings across studies and draw meaningful comparisons. For example, while some studies rely on observational coding, others use physiological measures or self-report scales, making it difficult to establish a unified framework for evaluating tactile engagement.

Third, the absence of rigorous experimental designs—such as randomized controlled trials (RCTs) or long-term follow-up studies—limits our understanding of the sustained therapeutic effects of tactile interaction with SARs. Most existing studies are exploratory or short-term in nature, which restricts conclusions about long-term benefits or clinical efficacy.

Fourth, the review was limited to English-language publications, which may have introduced a linguistic bias and excluded relevant research conducted in other languages.

To address these gaps, recent studies have begun exploring innovative approaches. For instance, Ref. [80] introduced vibrotactile heartbeats in the PARO robot to enhance emotional regulation and perceived social presence, suggesting new directions for tactile design in SARs. Similarly, Ref. [83] demonstrated that affective tactile interaction with social robots can influence stress levels, trust, and even risk-taking behavior, highlighting the importance of touch as a social and emotional cue in HRI. The authors of [10] emphasized the need for standardized methodologies and categorized SAR roles (e.g., motivation, imitation, feedback) to better structure future research in rehabilitation contexts.

Moving forward, it is essential that future studies adopt methodologically robust designs, including larger and more diverse samples, standardized assessment frameworks, and longitudinal evaluations. These improvements will be critical for validating the therapeutic effectiveness of tactile interactions between children with physical disabilities and SARs, and for guiding the development of more effective, evidence-based robotic interventions.

### 4.7. Ethical Considerations

The clinical and therapeutic use of SARs in pediatric populations with physical disabilities raises important ethical considerations due to the vulnerability of this group. While many of the studies included in this review were experimental and conducted with appropriate ethical approvals, ethical standards and protocols vary across countries and institutions. These differences affect how consent is obtained, how safety is managed, and how the psychological and physical well-being of participants is protected.

This variability makes it challenging to provide a consistent ethical assessment of all studies. Moreover, touch-based interactions with semi-autonomous agents introduce additional concerns, such as respecting personal autonomy, maintaining appropriate physical boundaries, and preventing unintended consequences.

As SARs continue to be developed for pediatric rehabilitation, it is essential that future research explicitly incorporates ethical frameworks from the field of HRI. Doing so will help ensure that these technologies are implemented in ways that are safe, inclusive, and sensitive to the clinical and social contexts in which they are used.

## 5. Conclusions and Future Work

This review examined the role of tactile interaction in studies involving SARs for children with physical disabilities, such as CP. Our findings reveal a notable gap in the literature regarding the integration and evaluation of tactile interaction in therapeutic settings. The following summarizes our responses to the four research questions (RQs):

1. RQ1: Have previous studies involving SARs in therapeutic settings for children with physical disabilities (such as CP) incorporated tactile interaction? Yes, but only partially. Among the nine eligible studies, only four explicitly evaluated tactile interaction, indicating that this modality is underrepresented in SAR-based therapy research.
2. RQ2: Is it possible to engage SARs and children with physical disabilities (such as CP) in meaningful touch-based interactions? In what ways does this affect the outcomes of therapy? The reviewed studies demonstrate that meaningful tactile interaction is not only possible but also beneficial. Children who engaged in touch-based interactions with SARs showed increased motivation, more frequent task repetition, and richer social responses—factors that positively influence therapeutic outcomes.
3. RQ3: How does the current literature describe and measure the duration, impact, and effectiveness of tactile interactions between SARs and children with physical disabilities? The literature lacks consistency in how tactile interactions are described and measured. While some studies used behavioral observations or qualitative feedback, few employed standardized or quantitative methods. This highlights the need for multimodal evaluation protocols that combine behavioral, physiological, and self-report data to assess tactile engagement more rigorously.
4. RQ4: What are the key findings from the studies that included touch or tactile interactions with SARs in therapeutic settings? Key findings suggest that tactile interaction can enhance therapy by fostering emotional connection, improving engagement, and supporting therapeutic goals. However, small sample sizes and limited methodological consistency restrict the generalizability of these results.

The SARs reviewed in this study were designed to support a variety of therapeutic goals tailored to the needs of children with physical disabilities. These included mobility therapy, which focuses on encouraging physical movement and the development of motor skills; social therapy, aimed at enhancing social interaction, emotional expression, and overall engagement; and phonetic and communication therapy, which supports the development of speech and language abilities. Each of these therapeutic domains leverages the interactive capabilities of SARs to foster meaningful engagement and promote therapeutic progress in children with conditions such as cerebral palsy.

To advance the field, future research should focus on developing SARs with soft, responsive materials and touch-sensitive technologies that enable safe and engaging tactile interactions. Larger sample sizes and standardized evaluation methods are essential. Integrating behavioral, physiological, and self-report metrics into multimodal protocols will allow for more robust comparisons and facilitate the translation of promising findings into clinical practice and HRI research.

## Figures and Tables

**Table 1:** The sources used for searching publications.

Sources	Number of Publications	Period
ACM Digital Library	60	2010–2024
Springer	103	2010–2024
Google Scholar	69	2010–2024

**Table 4:** Information reported on robot platforms appearing in nine eligible studies.

Robot Platform	Tactile Sensors	Audio (Microphone/Speaker)	Video/Vision	Presence/Proximity	Other On-Board Sensors	Controller/Board
Modified Lego-Puppet [52]	2 × touch sensors (arms), 1 × ultrasonic (leg)	N/R	N/R	Ultrasonic (leg)	3 × smart-servo motors	Lego Mindstorms NXT Brick (Robolab 3)
NAO/ZORA (humanoid) [53,61]	2 × head, hand & bumper tactiles	✓	2 × HD camera	SONAR, IR receiver	9-axis IMU + foot FSR	Proprietary Intel-Atom board (Aldebaran)
IROMEC (mobile play robot) [54,57]	3 × Bluetooth buttons	✓	Body 13 inch & head 8 inch LCDs, Colour-tracking cam	13 ultrasound, 18 IR, Laser scanner	Wheel encoders	Robosoft proprietary controller; MCU details
Semiautonomous-SAR [56]	None (stuffed exterior for safe touch)	✓	Not reported (N/R)	1 × SONAR	Battery monitor	m3pi MCU + Wixel 2.4 GHz radio; remote server laptop handles Kinect & Xbox pad
GoBot (TurtleBot2 base) [55]	None	✓	RealSense RGB-D camera	Lidar on TurtleBot2	Inertial unit	TurtleBot2 base, Raspberry Pi 4 running ROS Noetic
Shelbytron (wheel-dog) [55]	None	✓	N/R	IR distance	LEDs	Teensy 3.6 MCU
SPELTRA [62]	Resistive 5-pt touchscreen	✓	5 MP cam	N/R	5 × servo joints	Raspberry Pi 2 Model B+
Huggable (teddy-bear) [63]	12 × capacitive touch, 2 × pressure pads	✓	Phone camera	Accelerometer/gyroscope in phone	12-DOF joint encoders	Android smartphone + SparkFun IOIO board

## Data Availability

The data presented in this study are available on request from the lead author.

## References

[1] Centers for Disease Control and Prevention (2024). Data and Statistics for Cerebral Palsy. https://archive.cdc.gov/www_cdc_gov/ncbddd/cp/data.html.

[2] Olusanya B.O., Gladstone M., Wright S.M., Hadders-Algra M., Boo N.Y., Nair M.K.C., Almasri N., Kancherla V., Samms-Vaughan M.E., Kakooza-Mwesige A. (2022). Cerebral palsy and developmental intellectual disability in children younger than 5 years: Findings from the GBD-WHO Rehabilitation Database 2019. Front. Public Health.

[3] Bayón C., Raya R., Lara S.L., Ramirez O., Serrano J., Rocon E. (2016). Robotic therapies for children with cerebral palsy: A systematic review. Transl. Biomed..

[4] Rosenbaum P., Paneth N., Leviton A., Goldstein M., Bax M., Damiano D., Dan B., Jacobsson B. (2007). A report: The definition and classification of cerebral palsy April 2006. Dev. Med. Child Neurol. Suppl..

[5] McIntyre S., Morgan C., Walker K., Novak I. (2011). Cerebral palsy—Don’t delay. Dev. Disabil. Res. Rev..

[6] Adlakha S., Chhabra D., Shukla P. (2020). Effectiveness of gamification for the rehabilitation of neurodegenerative disorders. Chaos Solitons Fractals.

[7] Paulson A., Vargus-Adams J. (2017). Overview of Four Functional Classification Systems Commonly Used in Cerebral Palsy. Children.

[8] Trost M.J., Ford A.R., Kysh L., Gold J.I., Matarić M. (2019). Socially assistive robots for helping pediatric distress and pain: A review of current evidence and recommendations for future research and practice. Clin. J. Pain.

[9] Winkle K., Caleb-Solly P., Turton A., Bremner P. Social robots for engagement in rehabilitative therapies: Design implications from a study with therapists. Proceedings of the 2018 ACM/IEEE International Conference on Human-Robot Interaction.

[10] Carnevale A., Raso A., Antonacci C., Mancini L., Corradini A., Ceccaroli A., Casciaro C., Candela V., de Sire A., D’Hooghe P. (2025). Exploring the Impact of Socially Assistive Robots in Rehabilitation Scenarios. Bioengineering.

[11] Fernández-Batanero J.M., Montenegro-Rueda M., Fernández-Cerero J., García-Martínez I. (2022). Assistive technology for the inclusion of students with disabilities: A systematic review. Educ. Technol. Res. Dev..

[12] Li J. (2015). The benefit of being physically present: A survey of experimental works comparing copresent robots, telepresent robots and virtual agents. Int. J. Hum.-Comput. Stud..

[13] Malik N.A., Hanapiah F.A., Rahman R.A.A., Yussof H. (2016). Emergence of socially assistive robotics in rehabilitation for children with cerebral palsy: A review. Int. J. Adv. Robot. Syst..

[14] Bailey-Van Kuren M. (2007). Robotic solutions in pediatric rehabilitation. Rehabilitation Robotics.

[15] Ros R., Nalin M., Wood R., Baxter P., Looije R., Demiris Y., Belpaeme T., Giusti A., Pozzi C. Child-robot interaction in the wild: Advice to the aspiring experimenter. Proceedings of the 13th International Conference on Multimodal Interfaces.

[16] Butchart J., Harrison R., Ritchie J., Martí F., McCarthy C., Knight S., Scheinberg A. (2021). Child and parent perceptions of acceptability and therapeutic value of a socially assistive robot used during pediatric rehabilitation. Disabil. Rehabil..

[17] McCarthy C., Butchart J., George M., Kerr D., Kingsley H., Scheinberg A.M., Sterling L. Robots in rehab: Towards socially assistive robots for paediatric rehabilitation. Proceedings of the Annual Meeting of the Australian Special Interest Group for Computer Human Interaction.

[18] Jafari N., Adams K.D., Tavakoli M. (2016). Haptics to improve task performance in people with disabilities: A review of previous studies and a guide to future research with children with disabilities. J. Rehabil. Assist. Technol. Eng..

[19] Garcês Costa D.L., Chisik Y., dos Santos Faria A.L. (2018). Hugvie as a therapeutic agent in the improvement of interaction skills in children with developmental disabilities: An exploratory study. Proceedings of the Advances in Computer Entertainment Technology: 14th International Conference, ACE 2017.

[20] Costa S., Lehmann H., Dautenhahn K., Robins B., Soares F. (2015). Using a humanoid robot to elicit body awareness and appropriate physical interaction in children with autism. Int. J. Soc. Robot..

[21] Burns R.B., Seifi H., Lee H., Kuchenbecker K.J. (2020). Getting in touch with children with autism: Specialist guidelines for a touch-perceiving robot. Paladyn, J. Behav. Robot..

[22] Robins B., Amirabdollahian F., Ji Z., Dautenhahn K. (2010). Tactile interaction with a humanoid robot for children with autism: A case study analysis involving user requirements and results of an initial implementation. Proceedings of the 19th International Symposium in Robot and Human Interactive Communication.

[23] Robins B., Dautenhahn K., Boekhorst R.T., Billard A. (2005). Robotic assistants in therapy and education of children with autism: Can a small humanoid robot help encourage social interaction skills?. Univers. Access Inf. Soc..

[24] Pakkar R., Clabaugh C., Lee R., Deng E., Mataricć M.J. (2019). Designing a socially assistive robot for long-term in-home use for children with autism spectrum disorders. Proceedings of the 2019 28th IEEE International Conference on Robot and Human Interactive Communication (RO-MAN).

[25] Zheng X., Shiomi M., Minato T., Ishiguro H. (2019). What kinds of robot’s touch will match expressed emotions?. IEEE Robot. Autom. Lett..

[26] Van Erp J.B., Toet A. (2015). Social touch in human–computer interaction. Front. Digit. Humanit..

[27] Paterson M. (2023). Inviting robot touch (by design). ACM Trans. Hum.-Robot Interact..

[28] Saunderson S., Nejat G. (2019). How robots influence humans: A survey of nonverbal communication in social human–robot interaction. Int. J. Soc. Robot..

[29] Yohanan S.J. (2012). The Haptic Creature: Social Human-Robot Interaction Through Affective Touch. Ph.D. Thesis.

[30] Willemse C.J., Van Erp J.B. (2019). Social touch in human–robot interaction: Robot-initiated touches can induce positive responses without extensive prior bonding. Int. J. Soc. Robot..

[31] Sin M.T.A., Koole S.L. (2013). That human touch that means so much: Exploring the tactile dimension of social life. Mind Mag..

[32] Willemse C.J., Toet A., Van Erp J.B. (2017). Affective and behavioral responses to robot-initiated social touch: Toward understanding the opportunities and limitations of physical contact in human–robot interaction. Front. ICT.

[33] Silvera-Tawil D., Rye D., Velonaki M. (2015). Artificial skin and tactile sensing for socially interactive robots: A review. Robot. Auton. Syst..

[34] Umeda N., Ishihara H., Ikeda T., Asada M. (2022). The first impressions of small humanoid robots modulate the process of how touch affects personality what they are. Adv. Robot..

[35] Yasaman S. (2015). Design and evaluation of a touch-centered calming interaction with a social robot. IEEE Trans. Affect. Comput..

[36] Teyssier M., Bailly G., Pelachaud C., Lecolinet E. (2020). Conveying emotions through device-initiated touch. IEEE Trans. Affect. Comput..

[37] Zhou Y., Kornher T., Mohnke J., Fischer M.H. (2021). Tactile interaction with a humanoid robot: Effects on physiology and subjective impressions. Int. J. Soc. Robot..

[38] Block A.E., Kuchenbecker K.J. (2019). Softness, warmth, and responsiveness improve robot hugs. Int. J. Soc. Robot..

[39] Shiomi M., Nakagawa K., Shinozawa K., Matsumura R., Ishiguro H., Hagita N. (2017). Does a robot’s touch encourage human effort?. Int. J. Soc. Robot..

[40] Bock N., Hoffmann L., Rosenthal-vd Pütten A. Your Touch Leaves Me Cold, Robot. Proceedings of the Companion of the 2018 ACM/IEEE International Conference on Human-Robot Interaction.

[41] Shiomi M., Nakata A., Kanbara M., Hagita N. (2017). A hug from a robot encourages prosocial behavior. Proceedings of the 2017 26th IEEE International Symposium on Robot and Human Interactive Communication (RO-MAN).

[42] Avelino J., Correia F., Catarino J., Ribeiro P., Moreno P., Bernardino A., Paiva A. (2018). The power of a hand-shake in human-robot interactions. Proceedings of the 2018 IEEE/RSJ International Conference on Intelligent Robots and Systems (IROS).

[43] Csala E., Németh G., Zainko C. (2012). Application of the NAO humanoid robot in the treatment of marrow-transplanted children. Proceedings of the 2012 IEEE 3rd International Conference on Cognitive Infocommunications (CogInfoCom).

[44] Pulido J.C., Suarez-Mejias C., Gonzalez J.C., Ruiz A.D., Ferri P.F., Sahuquillo M.E.M., De Vargas C.E.R., Infante-Cossio P., Calderon C.L.P., Fernandez F. (2019). A socially assistive robotic platform for upper-limb rehabilitation: A longitudinal study with pediatric patients. IEEE Robot. Autom. Mag..

[45] Chen Y., Garcia-Vergara S., Howard A.M. (2018). Effect of feedback from a socially interactive humanoid robot on reaching kinematics in children with and without cerebral palsy: A pilot study. Dev. Neurorehabilit..

[46] Kachmar O., Kozyavkin V., Ablikova I. Humanoid social robots in the rehabilitation of children with cerebral palsy. Proceedings of the REHAB 2014.

[47] Gnjatović M., Tasevski J., Mišković D., Savić S., Borovac B., Mikov A., Krasnik R. (2017). Pilot corpus of child-robot interaction in therapeutic settings. Proceedings of the 2017 8th IEEE International Conference on Cognitive Infocommunications (CogInfoCom).

[48] Malik N.A., Yussof H., Hanapiah F.A., Rahman R.A.A., Basri H.H. (2015). Human-robot interaction for children with cerebral palsy: Reflection and suggestion for interactive scenario design. Procedia Comput. Sci..

[49] Gallace A., Spence C. (2010). The science of interpersonal touch: An overview. Neurosci. Biobehav. Rev..

[50] Dahiya R., Metta G., Valle M., Sandini G. (2010). Tactile Sensing—From Humans to Humanoids. IEEE Trans. Robot..

[51] Dario P. (1991). Tactile sensing: Technology and applications. Sens. Actuators A Phys..

[52] Hsieh H.C., Liu C.K., Chen P.K.H. (2020). Lego robots in puppet play for children with cerebral palsy. Proceedings of the Universal Access in Human-Computer Interaction. Design Approaches and Supporting Technologies: 14th International Conference, UAHCI 2020, Held as Part of the 22nd HCI International Conference, HCII 2020.

[53] van den Heuvel R.J., Lexis M.A., de Witte L.P. (2020). ZORA robot based interventions to achieve therapeutic and educational goals in children with severe physical disabilities. Int. J. Soc. Robot..

[54] Klein T., Gelderblom G.J., de Witte L., Vanstipelen S. (2011). Evaluation of short term effects of the IROMEC robotic toy for children with developmental disabilities. Proceedings of the 2011 IEEE International Conference on Rehabilitation Robotics.

[55] Helmi A., Wang T.H., Logan S.W., Fitter N.T. (2023). Harnessing the Power of Movement: A Body-Weight Support System & Assistive Robot Case Study. Proceedings of the 2023 International Conference on Rehabilitation Robotics (ICORR).

[56] Clark C., Sliker L., Sandstrum J., Burne B., Haggett V., Bodine C. (2019). Development and preliminary investigation of a semiautonomous Socially Assistive Robot (SAR) designed to elicit communication, motor skills, emotion, and visual regard (engagement) from young children with complex cerebral palsy: A pilot comparative trial. Adv. Hum.-Comput. Interact..

[57] Van Den Heuvel R.J., Lexis M.A., Janssens R.M., Marti P., De Witte L.P. (2017). Robots supporting play for children with physical disabilities: Exploring the potential of IROMEC. Technol. Disabil..

[58] Beets M.W., von Klinggraeff L., Weaver R.G., Armstrong B., Burkart S. (2021). Small studies, big decisions: The role of pilot/feasibility studies in incremental science and premature scale-up of behavioral interventions. Pilot Feasibility Stud..

[59] Paterson M. (2023). Social robots and the futures of affective touch. Senses Soc..

[60] Argall B.D., Billard A.G. (2010). A survey of tactile human–robot interactions. Robot. Auton. Syst..

[61] Martí Carrillo F., Butchart J., Knight S., Scheinberg A., Wise L., Sterling L., McCarthy C. (2018). Adapting a general-purpose social robot for paediatric rehabilitation through in situ design. ACM Trans. Hum.-Robot Interact. (THRI).

[62] Robles-Bykbaev V., Ochoa-Guaraca M., Carpio-Moreta M., Pulla-Sánchez D., Serpa-Andrade L., López-Nores M., García-Duque J. Robotic assistant for support in speech therapy for children with cerebral palsy [Conferencia]. Proceedings of the 2016 IEEE International Autumn Meeting on Power, Electronics and Computing (ROPEC 2016).

[63] Jeong S., Santos K.D., Graca S., O’Connell B., Anderson L., Stenquist N., Fitzpatrick K., Goodenough H., Logan D., Weinstock P. Designing a socially assistive robot for pediatric care. Proceedings of the 14th International Conference on Interaction Design and Children.

[64] Hoffmann L., Krämer N.C. (2021). The persuasive power of robot touch. PLoS ONE.

[65] Miguel Cruz A., Rios Rincon A.M., Rodriguez Dueñas W.R., Quiroga Torres D.A., Bohórquez-Heredia A.F. (2017). What does the literature say about using robots on children with disabilities?. Disabil. Rehabil. Assist. Technol..

[66] Blankenship M.M., Bodine C. (2020). Socially assistive robots for children with cerebral palsy: A meta-analysis. IEEE Trans. Med Robot. Bionics.

[67] Zhang G., Hansen J.P. (2022). Telepresence robots for people with special needs: A systematic review. Int. J. Hum.-Interact..

[68] Fridin M., Belokopytov M. (2014). Robotics agent coacher for cp motor function (rac cp fun). Robotica.

[69] Buitrago J.A., Bolaños A.M., Caicedo Bravo E. (2020). A motor learning therapeutic intervention for a child with cerebral palsy through a social assistive robot. Disabil. Rehabil. Assist. Technol..

[70] Céspedes N., Raigoso D., Múnera M., Cifuentes C.A. (2021). Long-term social human-robot interaction for neurorehabilitation: Robots as a tool to support gait therapy in the pandemic. Front. Neurorobot..

[71] Xu J., De’Aira G.B., Chen Y.P., Howard A. (2018). Robot therapist versus human therapist: Evaluating the effect of corrective feedback on human motor performance. Proceedings of the 2018 International Symposium on Medical Robotics (ISMR).

[72] De’Aira G.B., Xu J., Chen Y.P., Howard A. (2019). The effect of robot vs. human corrective feedback on children’s intrinsic motivation. Proceedings of the 2019 14th ACM/IEEE International Conference on Human-Robot Interaction (HRI).

[73] Bonarini A., Garzotto F., Gelsomini M., Romero M., Clasadonte F., Yilmaz A.N.Ç. (2016). A huggable, mobile robot for developmental disorder interventions in a multi-modal interaction space. Proceedings of the 2016 25th IEEE International Symposium on Robot and Human Interactive Communication (RO-MAN).

[74] Robins B., Dautenhahn K. (2014). Tactile interactions with a humanoid robot: Novel play scenario implementations with children with autism. Int. J. Soc. Robot..

[75] Robins B., Dautenhahn K., Dickerson P. (2012). Embodiment and cognitive learning–can a humanoid robot help children with autism to learn about tactile social behaviour?. Proceedings of the Social Robotics: 4th International Conference, ICSR 2012.

[76] Amirabdollahian F., Robins B., Dautenhahn K., Ji Z. (2011). Investigating tactile event recognition in child-robot interaction for use in autism therapy. Proceedings of the 2011 Annual International Conference of the IEEE Engineering in Medicine and Biology Society.

[77] Robins B., Amirabdollahian F., Dautenhahn K. Investigating child-robot tactile interactions: A taxonomical classification of tactile behaviour of children with autism towards a humanoid robot. Proceedings of the Sixth International Conference on Advances in Computer–Human Interactions (Citeseer).

[78] Krichmar J.L., Chou T.S. A Tactile Robot for Developmental Disorder Therapy. Proceedings of the Technology, Mind, and Society.

[79] Cansev M.E., Miller A.J., Brown J.D., Beckerle P. (2024). Implementing social and affective touch to enhance user experience in human-robot interaction. Front. Robot. AI.

[80] Borgstedt J., Macdonald S., Marky K., Pollick F.E., Brewster S.A. (2024). Soothing Sensations: Enhancing Interactions with a Socially Assistive Robot through Vibrotactile Heartbeats. Proceedings of the 2024 33rd IEEE International Conference on Robot and Human Interactive Communication (ROMAN).

[81] Guo F., Fang C., Ren Z., Li M. (2024). Potential applications of humanoid robotic touch for social regulation of emotion: Evidence from ECG and fNIRS. Behaviour & Information Technology.

[82] Lopes S., Magalhães P., Pereira A., Martins J., Magalhães C., Chaleta E., Rosário P. (2018). Games used with serious purposes: A systematic review of interventions in patients with cerebral palsy. Front. Psychol..

[83] Ren Q., Belpaeme T. (2024). Tactile Interaction with Social Robots Influences Attitudes and Behaviour. Int. J. Soc. Robot..

